# Fungal Infections Are Not Associated with Increased Mortality in COVID-19 Patients Admitted to Intensive Care Unit (ICU)

**DOI:** 10.1155/2023/4037915

**Published:** 2023-09-09

**Authors:** James Ainsworth, Peter Sewell, Sabine Eggert, Keith Morris, Suresh Pillai

**Affiliations:** ^1^Ed Major Intensive Care Unit, Morriston Hospital, Swansea, UK; ^2^Cardiff Metropolitan University, Cardiff, UK

## Abstract

**Introduction:**

Fungal infection is a cause of increased morbidity and mortality in intensive care patients. Critically unwell patients are at increased risk of developing invasive fungal infections. COVID-19 patients in the intensive care unit (ICU) may be at a particularly high risk. The primary aim of this study was to establish the incidence of secondary fungal infections in patients admitted to the ICU with COVID-19. Secondary aims were to investigate factors that may contribute to an increased risk of fungal infections and to calculate the mortality between fungal and nonfungal groups.

**Methods:**

We undertook a retrospective observational study in a tertiary ICU in Wales, United Kingdom. 174 patients admitted with COVID-19 infection from March 2020 until May 2021 were included. Data were collected through a retrospective review of patient's clinical notes and microbiology investigation results obtained from the online clinical portal.

**Results:**

81/174 (47%) COVID-19 patients developed fungal infections, 93% of which were Candida species, including *Candida albicans* (88%), and 6% had an Aspergillus infection. Age and smoking history did not appear to be contributing factors. The nonfungal group had a significantly higher body mass index (33 ± 8 vs. 31 ± 7, *p*=0.01). The ICU length of stay (23 (1–116) vs. 8 (1–60), *p* < 0.001), hospital length of stay (30 (3–183) vs. 15 (1–174) ± 7, *p* < 0.001), steroid days (10 (1–116) vs. 4 (0–28), *p*=0.02), and ventilation days (18 (0–120) vs. 2 (0–55), *p* < 0.001) were significantly higher in the fungal group. The mortality rate in both groups was similar (51% vs. 52%). The Kaplan–Meier survival analysis showed that the fungal group survived more than the nonfungal group (log rank (Mantel–Cox), *p* < 0.001).

**Conclusion:**

Secondary fungal infections are common in COVID-19 patients admitted to the ICU. Longer treatment with corticosteroids, increased length of hospital and ICU stay, and greater length of mechanical ventilation significantly increase the risk of fungal infections. Fungal infection, however, was not associated with an increase in mortality.

## 1. Introduction

Fungal infection is a cause of increased morbidity and mortality in intensive care patients [1]. Patients who develop secondary infections demonstrate worse outcomes, with prolonged ICU duration and hospital length of stay [1, 2]. Patients with COVID-19 in the intensive care unit (ICU) may be at particularly high risk of secondary fungal infections. With an increasing duration of stay in the intensive care unit, the risk of opportunistic fungal infections appears to increase [1]. In addition, steroids, particularly dexamethasone, are frequently used in the management of COVID-19, having been shown to improve mortality in COVID-19 [3]. However, the use of steroids has been shown to increase the prevalence of bacterial and fungal infections [1]. Broad-spectrum antibiotics are also frequently used in this population which further increases the risk of fungal infections [1]. Avoiding secondary or superadded hospital-acquired infections during a viral pandemic such as COVID-19 is extremely challenging and may be unavoidable, given the long durations of hospital stay and prolonged periods of mechanical ventilation, use of multiple broad-spectrum antibiotics, and treatments used in the primary condition such as steroids or other immunosuppressants [4]. A strong association between invasive ventilation and secondary infections has been demonstrated in other studies [2].

The occurrence of secondary bacterial infections following respiratory viral infection is well known, such as streptococcal pneumonia following influenza infection, which may be as high as 65% of cases [5]. The pathophysiology predisposing patients to secondary infection following viral illness is still poorly understood but may involve a number of functional and histological factors [6, 7]. Local tissue damage increases vulnerability to secondary infection and virus-mediated immunosuppression may have a key role [4, 8].

Critically ill patients such as those admitted to intensive care are at increased risk of developing invasive fungal infections. This may be due to a number of factors, such as the complexity of the underlying disease, comorbidities, indwelling lines, immunosuppression, use of multiple antibiotics, and mechanical ventilation [9]. The most common comorbidities seen are cardiovascular disease and diabetes mellitus [1]. Opportunistic secondary fungal infections also occur, capable of causing focal infection such as fungal pneumonia or invasive and/or disseminated infection [7]. Organisms such as *Aspergillus* and/or *Candida* are frequently seen [2, 10]. Delayed diagnosis, for example, due to the limited sensitivity of fungal diagnostic testing, and delayed or limited effective treatment may contribute to the high mortality. Prompt diagnosis and treatment are extremely important [1]. Similar to other viral infections such as influenza, secondary infection in COVID-19 patients is likely extremely common, with a rate suggested as high as 50% in patients who died as a result of COVID-19 [6]. The incidence and significance of secondary fungal infections in COVID-19 are less clear and perhaps less appreciated [6, 7]. COVID-19 patients may, however, be at a particularly high risk, with mortality in patients with COVID-19 and candidaemia as high as 80% in some cases [1].

The primary aim of this study was to establish the incidence of secondary fungal infections in patients admitted to the ICU with COVID-19. The secondary aim was to compare these patients (fungal group) with those without fungal infections (nonfungal group) and to investigate the factors that may have contributed to an increased risk of fungal infection, as well as mortality in the fungal and nonfungal groups.

## 2. Methods

### 2.1. Study Design and Patients

This retrospective study was undertaken in the adult ICU of Morriston Hospital, which is a tertiary teaching hospital in Wales, United Kingdom. The study recruited patients who were admitted to the ICU during the first and second waves of the pandemic from March 2020 until May 2021. The study did not require ethical approval, as advised by the Research and Development (R&D) department. The patients included were all >18 years old, and those patients who did not have a smoking history documented were excluded because this study was investigating the factors that contributed to the development of fungal infections. The patients were divided into two groups: one who developed fungal infection (fungal group) and another without a fungal infection (nonfungal group) based on the fungal growth in the sputum sample. Patient demographics, comorbidities, ICU length of stay (ICULOS), hospital length of stay (HLOS), body mass index (BMI), duration of treatment with steroids (steroid days), and duration of the patient receiving intubation and ventilation (ventilation days) were obtained from the clinical notes. The microbiology investigation results were obtained from the online clinical portal.

### 2.2. Statistical Analysis

All statistical analysis was carried out on an IBM Statistical Package for Social Sciences (SPSS) for Windows, version 28.0 (Armonk, NY: IBM Corp). The chi-square test was used to compare categorical variables. Continuous variables, if they were normally distributed, were reported as means and standard deviation (SD) or median and interquartile ranges (IQR) if they were not normally distributed. Categorical data were presented in absolute numbers (*n*) and in percentages (%). Comparisons were done with the Mann–Whitney *U* test for the median and IQR or a two-sample *t*-test for the mean and SD. The data were deemed significant when *P*  <  0.05. Survival analysis was performed using Kaplan–Meier method.

## 3. Results

### 3.1. Patient Recruitment

**Figure 1:**
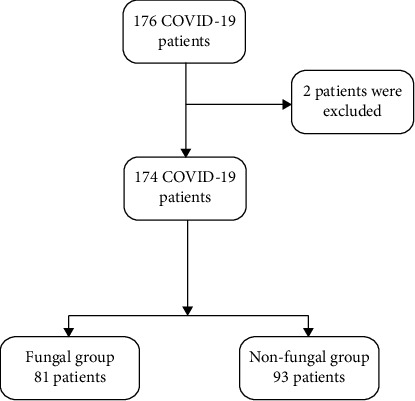
Flowchart of patients included in the study.

A total of 176 patients who were COVID-19 PCR-positive were admitted to the ICU. 2 patients were excluded because of their lack of smoking history. Therefore, this study included 174 patients with 81 patients in the fungal group and 93 patients in the nonfungal group (Figure 1).

### 3.2. Baseline Characteristics of the Groups

There was no statistical difference in age and gender; however, patients in the fungal group had a significantly higher BMI (*p*=0.01). The smoking history and comorbidities were not significantly different between the two groups. Patients in the fungal group had significantly higher ICULOS (*p* < 0.001), HLOS (*p* < 0.001), steroid days (*p*=0.02), and ventilation days (*p* < 0.001), as shown in Table 1. Patients who had steroids were placed on the standard dexamethasone 6 mg for 10 days either via a nasogastric tube or through an intravenous route.

**Figure 2:**
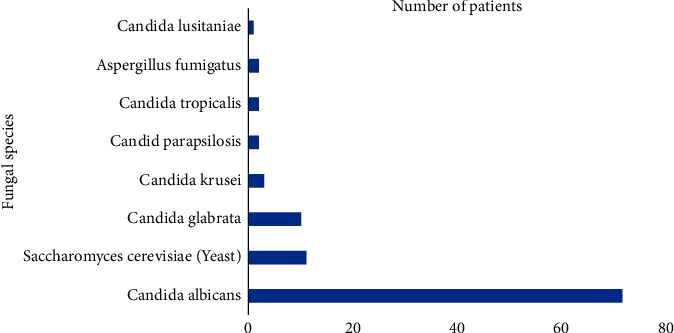
Bar chart showing fungal species grown in sputum and number of patients.

The sputum analysis of the patients in the fungal group showed that majority of them had grown *Candida albicans* (88%). About 20 (25%) patients grew more than one fungal species. Two (2.5%) patients grew *Aspergillus fumigatus*, 4 (5%) had serum *Aspergillus* antigen positive, and 2 (2.5%) were serum *Aspergillus* PCR positive. Patients who were either aspergillus antigen positive or PCR positive did not grow *Aspergillus* fungi in their sputum (Figure 2). All these patients were treated with either fluconazole, caspofungin, or amphotericin as advised by the microbiologist. All patients apart from one (who only had positive *Aspergillus* antigen on the serum sample) had a positive sputum sample (99%). 7 patients (9%) had positive blood samples. 8 patients (10%) had fungal isolates grown from central venous catheter tips. 10 patients (12%) had positive urine samples. Only 3 patients had recorded positive wound or skin swabs for fungal species. The mean days from ICU admission to confirmation of fungal infection were 7 days (including 8 patients who had confirmed positive fungal infection prior to or on the day of ICU admission, i.e., day 0). The mean days from hospital admission to confirmation of fungal infection were 10 days.

### 3.3. Survival Analysis

The Cox proportional hazards model was used to investigate the relationship between survival time and several predictor variables in the dataset (Table 2). The model showed that fungal infection (HR = 0.4053, *p*  <  0.001), age groups 41–60 (hazard ratio = 0.1940, *p*  <  0.001), and age groups 61–80 (hazard ratio = 0.2840, *p*=0.004) had significant associations with the hazard of the event, indicating higher survival probabilities for these groups. The model's concordance index was 0.702, suggesting reasonably good predictive performance. Other variables, such as sex, hypertension, DM, COPD, and asthma, did not show significant associations with the event.

**Figure 3:**
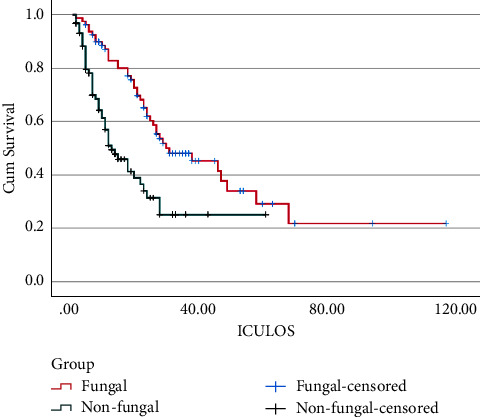
Kaplan–Meier survival analysis shows that the fungal group survived more than the nonfungal group (log rank (Mantel–Cox), *p* < 0.001).

There was no significant difference in the mortality between fungal and nonfungal groups (51% vs. 52%, *p*=0.90). The Kaplan–Meier survival analysis showed that fungal group survived more than the nonfungal group (log rank (Mantel–Cox), *p* < 0.001); therefore, fungal infections were not associated with increased mortality (Figure 3).

## 4. Discussion

This study aimed to determine the incidence of secondary fungal infections in patients admitted to our ICU with COVID-19. This study included 174 patients, all of whom were admitted to the ICU with PCR-confirmed COVID-19. Patients with confirmed fungal infection (fungal group) were then compared with those without fungal infection (nonfungal group), enabling us to investigate the factors most strongly associated with secondary fungal infection.

Secondary bacterial infection following respiratory viral illness is a well-documented and common occurrence, which complicates management, and contributes to increased morbidity and mortality [11, 12]. Similarly, secondary fungal infections also occur and may also lead to increased mortality [11, 13, 14]. Patients may be predisposed to secondary bacterial and fungal infections following viral illness due to the direct effects of the virus and/or combination of virus and drug-induced immunosuppression [15].

This study shows that fungal infections are common in COVID-19 patients admitted to our ICU, seen in almost half of patients (47%). The majority of fungal infections isolated were *Candida* species (93%), of which the most frequently seen was *Candida albicans. Candida albicans* was identified in 88% of cases. 25% of patients grew more than one fungal species. Other *Candida* species identified were as follows: *Candida glabrata* (12*%); Candida parapsilosis* (4%); *Candida krusei* (4%); *Candida tropicalis* (2%); and *Candida lusitaniae* (1%). Only 6 patients (7%) were positive for *Aspergillus* (*Aspergillus Fumigatus* isolated; *Aspergillus* PCR or antigen positive). This is consistent with other studies, showing the majority of secondary fungal infections in patients in hospitals and in ICUs with COVID-19 being *Candida albicans* [2, 16]. Fungal isolates in a cohort study in Brazil investigating secondary bacterial and fungal infections in patients with severe COVID-19 were 98.2% *Candida* spp. and 5.5% *Aspergillus* spp. [11]. An Egyptian cohort study also found *Candida* to be the most common species, present in 24.1% of cases, followed by *Aspergillus* (4.3%) [17]. A meta-analysis of 72 observational studies found that secondary bacterial infection was more prevalent than fungal infection, of which the most common organism identified was *Aspergillus* spp. The study also found that bacterial and fungal coinfections were more common in ICU-level care patients [12].

The mean days from ICU admission to confirmation of fungal infection were 7 days, with 8 patients having confirmed fungal infection on the day of or prior to ICU admission. The mean days from hospital admission to confirmation of a fungal infection was 10 days. This appears to be shorter than in other studies, with an average time from admission to positive culture data of 16.7 days (blood), 11.2 days (resp.), and 20.8 days (urine) in one study [3] and 19 days from admission to superinfection in another [14].

Various comorbidities have been shown to be associated with COVID-19, the most common of which is hypertension seen in 30% of patients, followed by diabetes and cardiovascular disease [18]. However, here, when investigating secondary fungal infections and COVID-19, comorbidities were not significantly different between the fungal and nonfungal groups, therefore, not appearing to contribute towards the risk of fungal infections in COVID-19 patients. Other studies have shown multiple comorbidities to be a risk factor for fungal coinfection [17]. The most common comorbidities seen across both groups were hypertension and diabetes, with minimal differences between the two. COPD and asthma were seen less frequently, again with no significant difference between the two groups.

Smoking is a primary cause of preventable disease worldwide and has a significant health burden [19]. Smoking impacts the immune system, and can increase the risk of infection, and it may contribute to increased morbidity and mortality in respiratory infections [19–21]. There has been conflicting evidence with regards to the association and impact of smoking and COVID-19 [21]. Smoking may, however, contribute to increased severity of disease or adverse outcomes and death. A meta-analysis of 47 studies using Medline, Embase, and Central suggested that smokers were at increased risk of severe disease and worse outcomes, including invasive ventilation and death [22]. In addition, smoking has been shown to be associated with increased invasive fungal disease [23]. Data on smoking history were collected and were included in the results of this study. Two patients were excluded due to a lack of smoking history, given the perceived potential for smoking status to act as a confounding factor.

Advanced age and male sex have both been shown to be associated with a higher risk of COVID-19 [11, 12]. The majority of patients in this study were male. Some studies have also stated that fungal infections were significantly associated with increased age [2, 11, 16], whereas others have found no significant difference in age, gender, or smoking habits [17]. Age and gender do not appear to have an impact on this study, with minimal differences between the two groups. Smoking history also does not alter the risk.

A number of factors were identified as having a strong association with secondary fungal infections in COVID-19 patients in the ICU. The factors contributing to a significant risk were: the duration of steroid treatment; the length of hospital and ICU stay; and duration of mechanical ventilation. Steroids have been shown to have a mortality benefit in COVID-19 and form part of the treatment guidelines, but concerns arise relating to the immunosuppressive nature of corticosteroids and the increased risk of secondary infections, particularly in critically unwell patients already at higher risk [3]. A cohort study of COVID-19 patients in an ICU in the United States with 147 patients found that 63% developed secondary infections and suggested that there was no significant increased risk of secondary infection in patients receiving corticosteroids [3]. Other studies have suggested a strong association between mechanical ventilation and the use of corticosteroids and the risk of fungal infection [2, 23]. Other immunosuppressive agents such as tocilizumab may also contribute to an increased risk of secondary bacterial and fungal infections [14]. In our study, a clear association is seen between longer treatment with corticosteroids and secondary fungal infections, with the mean number of steroid days in the fungal group greater than twice that of the nonfungal group (10 days vs 4 days).

Increased length of ICU stay and greater length of mechanical ventilation significantly increase the risk of fungal infections in COVID-19 patients in intensive care here, with a *p* value of less than 0.001. Both ICU and hospital length of stay were significantly longer in the fungal group compared with the nonfungal group, by a factor of greater than 100%. The mean hospital and ICU length of stay was 30 and 23 days in the fungal group and 15 and 8 in the nonfungal group.

The number of ventilation days in particular was significantly longer in the fungal group, with a mean of 18 days, compared with only 2 days in the nonfungal group. Fungal infection has been shown to be associated with increased mortality in some studies [16], with others demonstrating no increase in COVID-19 mortality [3]. In our study, the overall mortality in the fungal group was 51% and 52% in the nonfungal group. This suggests that fungal infections are not associated with an increased mortality. In fact, Kaplan–Meier analysis of the data showed that the fungal group survived more than the nonfungal group.

Our study supports much of the literature that fungal infections may represent an important complicating factor in patients admitted to the ICU with respiratory viral illnesses, such as COVID-19, and should be considered in all of these patients, particularly those with additional risk factors.

## 5. Conclusion

Secondary fungal infections are common in patients admitted to the ICU with COVID-19, seen in almost half of cases. *Candida* species was the most frequently isolated fungi. Male sex, age, and smoking history do not appear to confer a higher risk of secondary fungal infection. Longer treatment with corticosteroids, an increased length of ICU stay, and a greater duration of mechanical ventilation are significantly associated with secondary fungal infections. Reinforcing infection control policies and early consideration and screening for secondary fungal infection postviral illness such as COVID-19 may be important in avoiding delay in diagnosis and treatment. In addition, fungal infections were not associated with increased mortality in this study group.

### 5.1. Limitations

This was a retrospective, single-center study, which may, therefore, limit the generalisability of the results to other intensive care units and limit interpretation of the temporal or causal association between the development of secondary fungal infections and the associated factors investigated. There was no follow-up beyond the acute ICU admission with COVID-19; therefore, any data on longer-term survival or successful discharge from the hospital was unknown, particularly if those with secondary fungal infections are more likely to have a prolonged admission. Any conclusions regarding mortality should therefore be made cautiously.

## Figures and Tables

**Table 1:** Patient characteristics at baseline.

Variables	Fungal (*n* = 81)	Nonfungal (*n* = 93)	*p* value
Age (±SD)	59 ± 11	57 ± 12	0.27
Male gender	56 (70%)	56 (60%)	0.18
BMI (±SD)	33 ± 8	31 ± 7	0.01^*∗*^
Smoking			
Current smoker	6 (7%)	5 (5%)	0.58
Ex-smoker	27 (33%)	24 (26%)	0.28
Never smoker	48 (59%)	64 (70%)	0.19
Comorbidities			
Hypertension	32 (40%)	43 (46%)	0.37
Diabetes	18 (22%)	22 (24%)	0.82
COPD	3 (4%)	3 (3%)	0.86
Asthma	11 (14%)	8 (9%)	0.29
ICULOS (IQR)	23 (1–116)	8 (1–60)	<0.001^*∗*^
HLOS (IQR)	30 (3–183)	15 (1–174)	<0.001^*∗*^
Steroid days (IQR)	10 (1–116)	4 (0–28)	0.02^*∗*^
Ventilation days (IQR)	18 (0–120)	2 (0–55)	<0.001^*∗*^
Died	41 (51%)	48 (52%)	0.90

^*∗*^Indicates variables with statistical significance (*p*  < 0.05). ICULOS and HLOS are represented in days.

**Table 2:** Cox proportional hazards analysis results for factors influencing survival outcomes.

Variables	Hazard ratio	95% confidence interval	*p* value
Fungal infection	0.4053	(0.25672, 0.6397)	<0.001
Age 41–60	0.1940	(0.08059, 0.4669)	<0.001
Age 61–80	0.2840	(0.11983, 0.6731)	0.004
Age 81 and above	2.1972	(0.42846, 11.2675)	0.345
Sex (female)	1.1199	(0.70714, 1.7737)	0.629
Never smoked	1.7682	(1.08697, 2.8763)	0.022
Hypertension	0.9425	(0.60682, 1.4638)	0.792
DM	0.9473	(0.58452, 1.5351)	0.826
COPD	0.7463	(0.16834, 3.3084)	0.700
Asthma	0.9039	(0.45612, 1.7912)	0.772

Concordance index (CI) = 0.702; likelihood ratio test *p* value <0.001; Wald test *p* value <0.001; score (log rank) test *p* value <0.001.

## Data Availability

Access to data is restricted due to concerns relating to patient privacy.
